# Rare association of Visceral leishmaniasis with Hodgkin's disease: A case report

**DOI:** 10.1186/1750-9378-6-17

**Published:** 2011-10-20

**Authors:** Rakshit Kumar, Mradul K Daga, Nitish L Kamble, Arpit Sothwal, Tejinder Singh, Hemanta K Nayak, Nishant Raizada

**Affiliations:** 1 Department of Medicine, Maulana Azad Medical College, Bahadur Shah Zafar Marg, New Delhi, 110002, India; 2 Department of Pathology, Maulana Azad Medical College, Bahadur Shah Zafar Marg, New Delhi, 110002, India

**Keywords:** Visceral leishmaniasis, Hodgkin's disease

## Abstract

We present here a case of young male with complaints of fever and swelling in the neck for eight months. History of progressive weakness associated with weight loss was present. Physical examination revealed pallor, multiple enlarged cervical lymph nodes and hepatosplenomegaly. Investigations showed pancytopenia, hyperglobinemia and Leishman-Donovan bodies on bone marrow aspiration. Serological test confirmed diagnosis of visceral leishmaniasis. However, cervical lymph node aspiration and biopsy were suggestive of Mixed cellularity Hodgkin's disease. This made it a very rare case of Leishmaniasis as an opportunistic infection in a patient of pre-chemotherapy Hodgkin's disease. There was marked improvement in haematological profile and regression of hepatosplenomegaly with Amphotericin B treatment followed by favourable response to chemotherapy. The case emphasizes the suspicion for leishmaniasis as a masquerader and as an opportunistic infection in haematological malignancies.

## 

Leishmaniasis is frequently reported in patients with immunocompromised states due to HIV infection, post organ transplantation, solid tumors and chemotherapy [1,2]. However there are very few case reports of leishmaniasis with haematological malignancies, and even fewer with lymphoma, particularly Hodgkin's disease [3-6]. To the best of our knowledge, there are three case reports of leishmaniasis in post-chemotherapy Hodgkin's disease patients and only two case reports [7,8] of leishmaniasis in patients of Hodgkin's disease, pre-chemotherapy [9]. The case presented herein is a rare case of association of visceral leishmanisis and Hodgkin's disease right from the beginning and delves into the pathological and practical implications of their coexistence [10,11].

The patient, 18-year-old male, presented with a history of fever and multiple swellings in left side of neck for last eight months. Fever was moderate-to-high grade, intermittent, associated with sweating, without any chills or rigors. The patient noticed progressively enlarging swellings in the left side of neck which were painless, without any change on the overlying skin. In the last two months, the patient also experienced easy fatigability, weakness and swelling in both feet. Patient's father noticed weight loss which was not documented. The patient had been on irregular treatment from local practitioner without any relief in symptoms. The patient did not give any history of tuberculosis or any other systemic illness. There was no history of sexual contact, blood transfusion, or drug addiction and no significant family history was present.

On general physical examination, the patient was conscious, alert, and was of thin built. Vitals were stable and body temperature was 39°C. Marked pallor was present with pitting pedal edema. There were multiple enlarged cervical lymph nodes in posterior triangle of the neck on left side. Lymph nodes were firm in consistency, varied in size (2-4 cm), were discrete and were mobile, with normal overlying skin and temperature. On systemic examination, only mild hepatomegaly and moderate splenomegaly were seen.

Laboratory reports revealed pancytopenia with haemoglobin of 2.7 gm/dl, platelet count of 1.31 lacs, leucocyte count of 3500, ESR-70, and peripheral smear showed microcytic hypochromic anaemia. Total serum proteins were raised with albumin being low (2 gm/dl). Ultrasonography of abdomen revealed hepatomegaly (liver span 18 cm) and splenomegaly (splenic span 20 cm). Other investigations including chest X-ray, electrocardiography, viral markers and HIV serology were normal. CECT chest and abdomen revealed hepatosplenomegaly, minimal ascites and minimal pleural effusion. Echocardiography was essentially normal. FNAC of cervical lymph node was inconclusive and was repeated followed by excision biopsy. Meanwhile, bone marrow aspiration was done, which showed intracellular and extracellular Leishman-Donovan bodies (Figure 1). This was followed by a positive serology test for leishmaniasis. Patient was immediately started on Amphotericin B and given three units of blood transfusions. FNAC and excision biopsy reports confirmed the diagnosis of Mixed cellularity Hodgkin's disease (Figure 2 and 3), making it an exceptional coexisting pathology. Marked clinical and hematological improvement was seen, with regression of hepatosplenomegaly. The patient received three cycles of chemotherapy and showed positive response.

**Figure 1:**
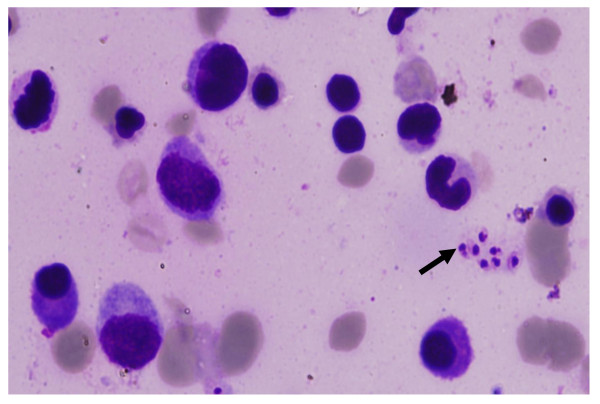
**Bone marrow slides showing Leishman-Donovan bodies (arrow)**.

**Figure 2:**
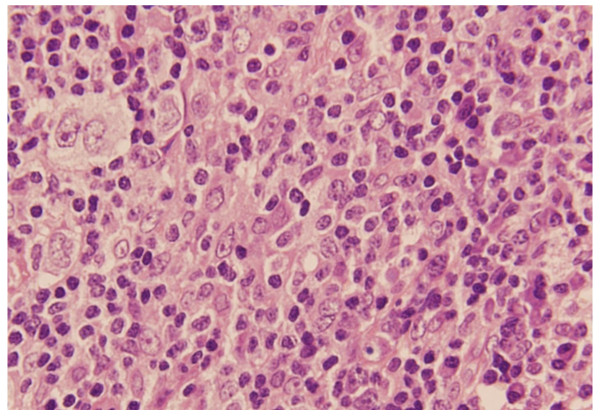
**Mixed cellularity seen in Hodgkin's disease**.

**Figure 3:**
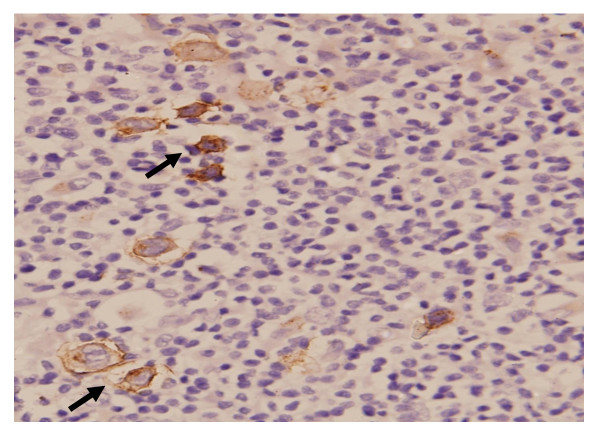
**Immunohistochemistry showing CD30 marker (arrow) in the lymph node**.

The clinical forms of leishmaniasis are visceral leishmaniasis (VL), cutaneous leishmaniasis (CL) and mucocutaneous leishmaniasis (ML). Out of 1.5-2 million infected patients worldwide, 90% of CL cases are in the endemic zone of Afghanistan, Pakistan, Middle East, Brazil and Peru. However, 90% of VL cases are seen to occur in the endemic zone of India, Bangladesh, Nepal, Sudan and Brazil. Cervical lymphadenopathy is uncommon in India though common in Mediterranean countries, Africa and China. On the other hand, incidence of malignancy is 11 million new cases per year, worldwide, with many cases occurring in the endemic zone of leishmaniasis. Moreover, the medical management of malignancy induces further immunosuppression, a condition which is already present in the setting of lymphoma and leukaemia, and becomes a breeding ground for opportunistic infections like leishmaniasis.

Leishmaniasis has emerged as a severe opportunistic infection in endemic areas. In immunocompromised hosts, it can be asymptomatic, often with no splenomegaly, and may be present in unusual location and run a severe and refractory course with frequent relapse. Although leishmaniasis and cancer are very common diseases, little attention has been paid to the pathophysiological and practical implications of their co-existence. Leishmaniasis is recognized as an opportunistic infection in HIV and malignancy patients [1,2], but reports with lymphoma are scarce [3-5]. There are three case reports of leishmaniasis in post chemotherapy Hodgkin's disease patients, including the recently published study on coexistence of both in a single lymph node, after multiple cycles of chemotherapy [6]. To the best of our knowledge, there are only two case reports [7,8] (Benhamou et al [7] and Magnan et al [8]) of leishmaniasis with pre-chemotherapy Hodgkin's disease, similar to our case.

In a significant review involving 37 case studies and 44 patients, Kopterides et al (2007)[9] found four types of association between leishmaniasis and malignant disorders: 1) Leishmaniasis masquerading as a malignant disorder, 2) Leishmaniasis developing as a difficult-to-diagnose/treat infection among patients receiving chemotherapy for various malignant disorders; 3) Simultaneous diagnosis of leishmaniasis and a neoplastic disorder in the same tissue samples of immunocompromised patients, and 4) Direct involvement of Leishmania spp. in the pathogenesis of cancerous lesions. Mangoud et al [10] reported that dysplasia was detected surrounding the leishmanial ulcer in 5 of 35 CL cases. Leishmaniasis adversely affects the activation and function of macrophages and dendritic cells [11], permitting the escape of malignant cells from immune destruction. Further, chronic leishmanial infections cause CD4 lymphopenia and low CD4/CD8 ratio. Similarly, Hodgkin's/Reed-Stenberg cells induce regulatory T cells that impair adaptive immune responses by releasing IL-10 and TNF B. Both the cytokines block the activation of naive and effector T cells, and have been reported to suppress the immune response to leishmania. Hence, mutual immunomodulation of leishmaniasis and Hodgkin's disease is highlighted in this patient and requires further epidemiological and pathology studies to understand the exact association or causal relation.

## Consent

Written informed consent was obtained from the patient for publication of this case report. A copy of the written consent is available for review by the Editor-in-Chief of this journal.

## List of abbreviations

HIV: Human Immunodeficiency Virus; ESR: Erythrocyte Sedimentation Rate; CECT: Contrast Enhanced Computed Tomography; FNAC: Fine Needle Aspiration Cytology; CL: Cutaneous Leishmaniasis; IL-10: Interleukin-10; TNF B: Tumor Necrosis Factor Beta.

## Competing interests

The authors declare that they have no competing interests.

## Authors' contributions

RK: Conceptualization of the manuscript, search of literature, and drafting and writing the manuscript, head of the resident team managing the patient

MKD: Vital inputs in drafting and editing of the manuscript, supervised clinical management of the patient being the head of the medical unit

NLK: Helped in literature search, editing and proofreading the manuscript

AS: Helped in literature search, editing and proofreading the manuscript

TS: Provided pathological insights in the case, with his vast experience on past such cases

HKN: Part of the investigative team involved in clinical management

NR: Part of the investigative team involved in clinical management.

All authors read and approved the final manuscript.
